# Bilateral Congenital Nasolacrimal Duct Cyst: A Rare Cause of Nasal Obstruction

**DOI:** 10.7759/cureus.8742

**Published:** 2020-06-21

**Authors:** Manish Gupta, Habibulla Khan, Monica Gupta

**Affiliations:** 1 Otorhinolaryngology, Maharishi Markandeshwar Institute of Medical Sciences and Research, Ambala, IND; 2 Otorhinolaryngology, Maharishi Markandeshwar Institute of Medical Science and Research, Ambala, IND; 3 General Medicine, Government Medical College and Hospital, Chandigarh, IND

**Keywords:** nasolacrimal duct cyst, nasal obstruction, intra-nasal cyst, pediatric airway, dacryocystocele

## Abstract

Nasal obstruction in neonates often results in respiratory discomfort, as neonates are obligate nasal breathers. Congenital bilateral nasal obstruction is an emergency situation which is generally secondary to choanal atresia. Rarely bilateral nasolacrimal duct (NLD) cyst causing intranasal swelling may be the underlying etiology. Neonatal respiratory distress warrants immediate measures to secure the airway and prompt investigations to reach a diagnosis for a definitive management. We describe a case of two-day-old girl with intermittent breathing difficulty because of bilateral NLD cysts causing nasal obstruction. The patient improved with conservative medical management.

## Introduction

Congenital anomaly of nasolacrimal system is common, but dacryocystocele is an uncommon manifestation especially when it presents as bilateral intranasal cyst. The dacryocystocele is formed when there is concomitant proximal (valve of Rosenmuller) and distal (valve of Hasner) membranous obstruction of the nasolacrimal drainage system, thus causing lacrimal sac and/or duct distension [1,2]. The patient may present with bluish cystic swelling inferomedial to medial canthus externally or as intranasal inferior meatal mass [3].

Usually, the garden variety of dacryocystocele presents with unilateral facial swelling located inferomedial to the medial canthus of the eye. In the case of rare congenital intranasal cysts, dacryocystocele may extend intranasally forming a nasal cyst located in the inferior meatus and present predominantly with respiratory distress, especially if bilateral. Most patients have epiphora due to functional closure of Rosenmuller valve present at the superior end of the tear sac and failure to open Hasner valve at the distal end of the nasolacrimal duct (NLD) [4].

## Case presentation

A two-day-old girl, born as full-term normal vaginal delivery, was referred to our department for intermittent episodes of difficult breathing as noticed by her mother. She also complained that the child had noisy respiration while sleeping and had excessive tearing. The symptoms neither progressed nor relieved on crying or on making the baby prone. There was no history of breath holding, difficulty while suckling, or bluish discoloration of her skin. There were no antepartum or intrapartum complications; the Apgar score was 10/10 and the birth weight was adequate.

On examination, the baby was lying comfortably beside her mother and her respiratory rate was within normal limits (40/min). On closer examination, there was an intermittently occurring audible rush of air through the nasal passage during inspiration, more noticeable with the child’s mouth closed. There was mild epiphora and concomitant sticky mucopurulent eye discharge bilaterally. There were no signs of respiratory distress in the form of grunting, stertor, stridor, alar collapse, sternal or intercostal recession, and the oxygen saturation at room air was 97%. There were no episodes of apnea, cyanosis, or epistaxis. Importantly, there was no rhinorrhea or mucus impaction on nasal inspection. Additionally, there were no signs of any craniofacial dysmorphism, ear or nasal malformations, or any comorbidities. Cardiac and other systemic examinations were within normal limits.

Examination of the nasal cavity with an otoscope revealed lateral wall bulge inferolateral to inferior turbinate, almost touching nasal septum, bilaterally. A nasogastric tube could be negotiated through the nares, which ruled out choanal atresia. There was no facial swelling and eye examination by ophthalmologist was reported as normal.

**Figure 1:**
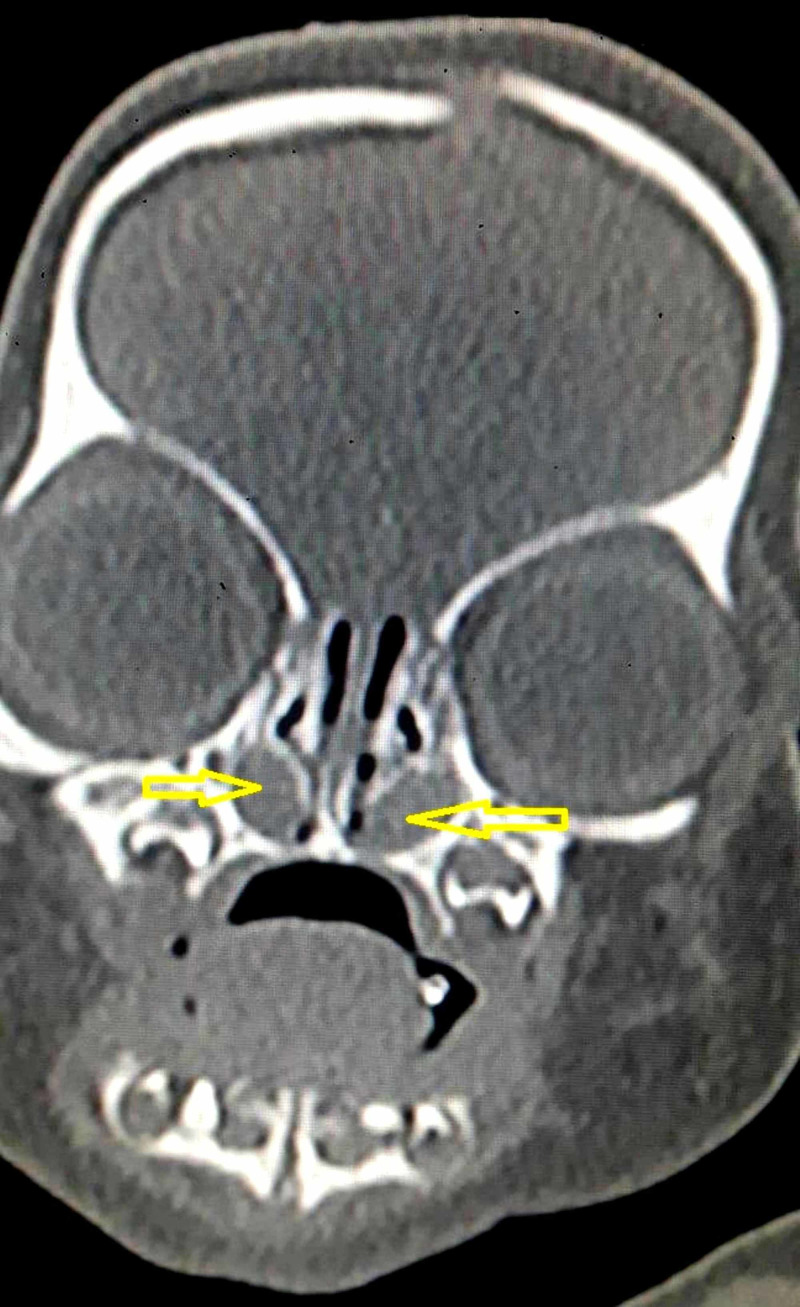
Non-contrast computed tomogram coronal section showing hypodense cystic mass in bilateral nasal cavity (yellow arrows), inferolateral to inferior turbinate.

**Figure 2:**
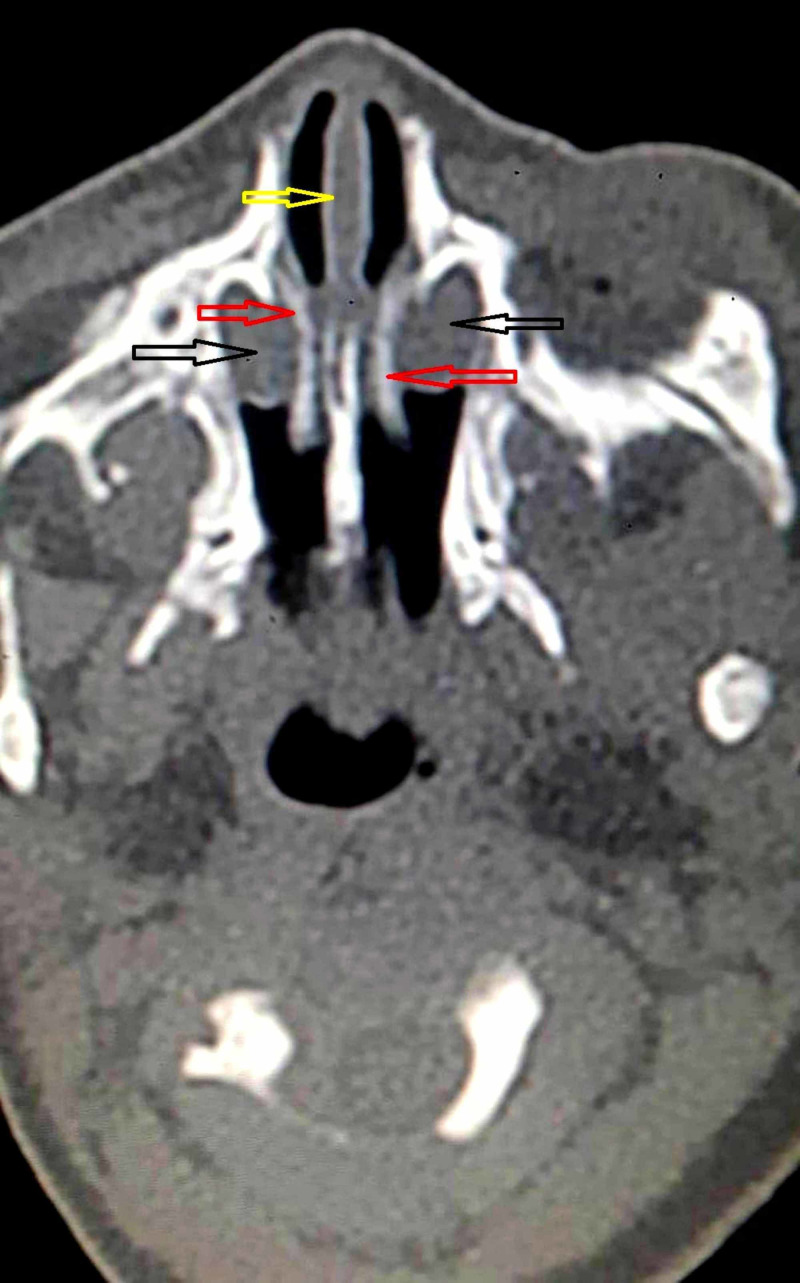
Non-contrast computed tomogram axial section showing intranasal cystic masses (black arrows) pushing inferior turbinates (red arrows) medially touching the nasal septum (yellow arrow) and completely obstructing bilateral nasal cavity.

Non-contrast computed tomogram (NCCT) of the nose and paranasal sinuses revealed hypodense cystic swellings in bilateral nasal cavities. The lesions were located inferior and lateral to inferior turbinates, pushing them medially in apposition with nasal septum (Figures 1, 2).

It was decided to give a conservative trial of warm compresses, nasal decongestants, antibiotic eye drops, and repeated lacrimal sac pressure and manual digital massage medial to medial canthus, towards the inferior direction. The patient responded well and was symptom free on the fifth day. The masses showed significant resolution and normal nasal cavity patency was noted on intranasal examination on the 10th day. 

## Discussion

Newborns are obligate nasal breathers until they are at least two months old. Thus, any nasal obstruction may have serious consequences and lead to respiratory distress. The most common cause of nasal obstruction is inflammatory edema; nevertheless, examination may reveal structural abnormalities, necessitating surgical intervention. The main surgical differential diagnosis is choanal atresia which is a congenital narrowing of the posterior nasal airway by a bony or membranous septum. In bilateral choanal atresia, respiratory distress in the neonate is relieved on crying, as it allows the baby to breathe through mouth. In laryngomalacia, the stridor worsens with crying, feeding, and lying in the supine position, whereas it improves in the prone position. Other differentials to be considered for congenital nasal obstruction are nasal trauma, such as septal hematomas and septal subluxation, dacryocystocele, meningoencephalocele, dermoid cyst, Thornwald’s cyst, hemangioma, pyriform aperture stenosis, nasal hypoplasia, and glioma [2].

Congenital defects in nasolacrimal drainage occur in 20% of newborns, but its severe form, i.e. dacryocystocele, is rarely observed. Its frequency is in two in 1,000 live births [5]. Approximately 90% of dacryocystoceles are unilateral, thus making bilateral presentation further rare, as in our case [5]. Literature suggests a higher incidence of congenital lacrimal pathology in female babies due to a narrower NLD compared to males [6]. A case report of monozygotic twins, having bilateral congenital dacryocystocele, had suggested some genetic basis for its familial predisposition and formation [7]. The exact genetic basis has not been elucidated yet due to the rarity of such an association.

Imaging with CT and MRI techniques are helpful in distinguishing lesions and identifying intracranial communication. CT is quick and easy, and its diagnostic features include triad of lacrimal sac cystic dilatation, NLD dilatation, and intranasal cystic mass that appear homogeneous, well-defined, thin walled with fluid attenuation [8]. CT is the imaging of choice although MRI has the advantage of characterizing the content of the lesion without radiation exposure. 

Asymptomatic dacryocystocele may be managed conservatively with lacrimal sac massage as most (96%) resolve spontaneously by one year of age [9]. Dacryocystitis, if evident by the presence of pain, fever, purulent discharge, and eye congestion, needs systemic antibiotics. Untreated dacrocystitis may complicate and lead to orbital and facial cellulitis [9]. If lacrimal sac distension is excessive, it may lead to narrowing of the lid fissure or corneal astigmatism resulting in anisometropic amblyopia and permanent canthal asymmetry [10].

Surgical interventions, such as lacrimal probing by an ophthalmologist and intranasal endoscopic cyst marsupialization by an otorhinolaryngologist, may be required in patients with large external or intranasal cysts who develop apnea or stridor [10]. Mechanical probing through the valve of Rosenmuller and irrigation decompresses the nasolacrimal drainage system proximally, while endoscopy identifies intranasal cyst and marsupialization decompresses the system distally [11]. This combined surgical intervention helps in restoring continuity of nasolacrimal system. In our case, there was no respiratory distress or features of dacryocystitis as highlighted above; therefore, the child was managed conservatively. Kuboi et al. reported one case of congenital bilateral dacryocystocele with respiratory difficulty which was managed conservatively with nasal continuous positive airway pressure [4].

## Conclusions

Congenital nasal anomalies as seen in our patient are uncommon yet potentially life-threatening reasons of upper airway obstruction in newborn and can result in neonatal respiratory distress. Through this two-day-old newborn baby’s case, we wish to highlight the importance of the broad differential diagnosis and structural abnormalities that dealing physicians should know so that appropriate referrals are done and treatment can be promptly initiated. Timely CT allows for optimal bony definition, and it is the diagnostic modality of choice. Management depends on the severity of distress, type, and location of the anomaly, the main goal being establishment of nasal patency. Fortunately, most infants often improve with conservative medical management as seen in our case.
